# Secondary Adrenal Insufficiency Presenting With Severe Hyponatremia in an Elderly Patient With Primary Aldosteronism Suggesting Underlying Autonomous Cortisol Secretion

**DOI:** 10.1002/ccr3.73303

**Published:** 2026-08-07

**Authors:** Minami Toda, Shunsuke Takahashi, Hitomi Saito, Honami Mori, Yutaka Kuroda, Akito Maeshima

**Affiliations:** ^1^ Department of Internal Medicine JCHO Saitama Northern Medical Center Saitama Japan; ^2^ Department of Nephrology and Hypertension Saitama Medical Center Saitama Japan

**Keywords:** adrenal gland neoplasms, adrenal insufficiency, hyponatremia, primary aldosteronism

## Abstract

Possible unrecognized cortisol autonomy in primary aldosteronism may suppress the hypothalamic–pituitary–adrenal axis. Under physiological stress, this may contribute to secondary adrenal insufficiency and severe hyponatremia. Clinicians should consider adrenal insufficiency in patients with primary aldosteronism presenting with unexplained clinical deterioration.

## 1 Introduction

Primary aldosteronism (PA) is a common cause of secondary hypertension characterized by autonomous aldosterone secretion [1, 2, 3]. In some patients, PA coexists with autonomous cortisol secretion (ACS), even in the absence of overt Cushingoid features [4, 5, 6]. Chronic mild cortisol excess associated with adrenal adenomas is currently referred to as mild autonomous cortisol secretion (MACS), a condition increasingly recognized as clinically relevant despite the absence of overt Cushingoid features. MACS may suppress the hypothalamic–pituitary–adrenal (HPA) axis, potentially impairing stress responsiveness and predisposing patients to adrenal insufficiency under physiological stress [7, 8, 9].

Herein, we report an elderly patient with long‐standing PA who developed severe hyponatremia and suspected secondary adrenal insufficiency during hospitalization, raising the possibility of underlying cortisol autonomy.

## 2 Case History/Examination

**FIGURE 1:**
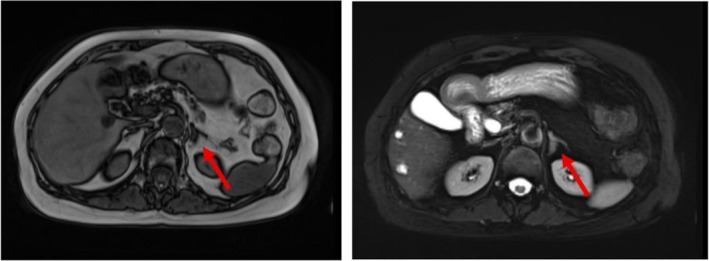
Abdominal MRI showing a left adrenal adenoma (arrow). A 7‐mm left adrenal mass was identified at the time of diagnosis of primary aldosteronism and showed no significant change in size or morphology during 13 years of follow‐up, indicating radiological stability.

An 80‐year‐old woman had been diagnosed with PA 13 years earlier. Imaging revealed a 7‐mm left adrenal adenoma. She declined adrenalectomy and was treated with spironolactone. Annual imaging showed no tumor progression (Figure 1).

She had experienced intermittent nausea and appetite loss for more than 10 years. Her medical history included diabetes mellitus, dyslipidemia, and angina pectoris. Her medications included aspirin, clopidogrel, vonoprazan, dapagliflozin, spironolactone, benidipine, etizolam, and macrogol. She had no history of glucocorticoid use.

On admission, her Glasgow Coma Scale score was E2V4M6, and her blood pressure was 98/68 mmHg. Her height was 149 cm, and her weight was 48.2 kg. No Cushingoid features, such as moon face, central obesity, or striae, were observed. Hypoglycemia was corrected; however, impaired consciousness persisted, leading to hospitalization.

Initial laboratory findings are summarized in Table 1. Mild hyponatremia (134 mEq/L) and hypoglycemia were present on admission, without evidence of severe organ dysfunction.

**TABLE 1:** Laboratory findings on admission.

CBC/Biochemistry			Urinalysis		
Parameter	Result	Reference range	Parameter	Result	Reference range
WBC	9100	3300–8900/μL	Specific gravity	1.025	1.000–1.050
Neut	64.8	35%–71%	pH	5	
Eosino	2.6	0%–6%	Protein	−	
Baso	0.6	0%–2%	Glucose	4+	
Mono	8.7	2%–10%	Ketone	2+	
Lympho	23.3	20%–51%	Occult blood	+	
RBC	490	360–489 × 10^4^/μL	Nitrite	−	
Hb	13.8	11.4–14.6 g/dL	RBC (sediment)	5–9	< 5/HPF
MCV	86.1	85–105 fL	WBC (sediment)	10–19	< 5/HPF
MCH	28.2	26–36 pg			
MCHC	32.7	30–35 g/dL			
Plt	13.2	14–35.9 × 10^4^/μL			
TP	5.7	6.5–8.0 g/dL			
Alb	3.6	4.0–5.0 g/dL			
T‐Bil	0.67	0.2–1.2 mg/dL			
AST	42	< 35 U/L			
ALT	29	< 35 U/L			
γGT	15	< 21 U/L			
T‐Cho	142	142–248 mg/dL			
LDL‐C	84	70–139 mg/dL			
TG	73	30–149 mg/dL			
CK	129	40–180 U/L			
BUN	13.8	9.0–22.0 mg/dL			
Cre	1.05	0.46–0.79 mg/dL			
eGFR	38.6	≥ 60 mL/min/L			
Na	134	135–145 mEq/L			
Cl	101	98–108 mEq/L			
K	3.9	3.5–5.0 mEq/L			
Glucose	43	70–109 mg/dL			
HbA1c	6.9	4.6%–5.6%			
CRP	3.47	< 0.3 mg/dL			

*Note:* Initial laboratory data demonstrated mild hyponatremia and hypoglycemia, without evidence of severe organ dysfunction.

## 3 Differential Diagnosis, Investigations and Treatment

During hospitalization, dapagliflozin was discontinued because of dehydration. On hospital day 10, her consciousness deteriorated further (E1V1M1). Laboratory findings at the time of clinical deterioration are summarized in Table 2. The patient developed severe hypotonic hyponatremia (serum sodium 113 mEq/L) with low plasma osmolality (231 mOsm/kg) and inappropriately concentrated urine (539 mOsm/kg).

**TABLE 2:** Laboratory findings at the time of clinical deterioration (hospital day 10).

CBC/Biochemistry			Endocrinal tests		
Parameter	Result	Reference range	Parameter	Result	Reference range
WBC	5300	/μL	ACTH	< 1.5	7.2–63.3 pg/mL
Neut	60.6	%	Cortisol	0.96	7.07–19.6 μg/dL
Eosino	8.5	%	PRA	2.0	0.2–2.3 ng/mL/h
Baso	0.9	%	PAC	215	4.0–82.1 pg/mL
Mono	12.9	%	AVP	2.3	< 2.8 pg/mL
Lympho	17.1	%	TSH	4.033	0.61–4.23 mIU/L
RBC	474	×10^4^/μL	FT3	1.09	2.24–3.94 pg/mL
Hb	13.5	g/dL	FT4	0.91	0.77–1.59 ng/dL
MCV	86.5	fL	HANP	12.3	0–43 pg/mL
MCH	28.5	pg	**Urinalysis**		
MCHC	28.5	g/dL	Specific gravity	1.015	
Plt	14.8	×10^4^/μL	pH	5.5	
TP	5.3	g/dL	Protein	±	
Alb	3.3	g/dL	Protein	15	mg/dL
T‐Bil	0.86	mg/dL	Glucose	+	
AST	33	U/L	Ketone	+	
ALT	18	U/L	Occult blood	−	
γGT	15	U/L	Nitrite	+	
UA	3	mg/dL	RBC (sediment)	1–4	/HPF
CK	45	U/L	WBC (sediment)	50–99	/HPF
BUN	6.6	mg/dL	Urine P/C ratio	0.15	mg/g·Cr
Cre	0.67	mg/dL	Urine OSM	539	mOsm/kgH_2_O
eGFR	63.2	mL/min/L	Urine Na	175	mEq/L
Na	113	mEq/L	Urine K	37	5–30 mEq/L
Cl	82	mEq/L	Urine Cl	148	100–200 mEq/L
K	3.8	mEq/L	Urine UA	30.1	10–100 mg/dL
Ca	8.3	mg/dL	Urine Cre	81.8	mg/dL
IP	3.5	mg/dL			
CRP	5.57	mg/dL			
Serum OSM	231	276–292 mOsm/kgH_2_O			
**VBG analysis**					
pH	7.45				
pCO2	30.1	mmHg			
pO2	43	mmHg			
Na^+^	114	mmol/L			
K^+^	3.7	mmil/L			
Cl^+^	80	mmol/L			
HCO_3_^−^	22.7	mmol/L			

*Note:* The patient developed severe hypotonic hyponatremia with low plasma osmolality and inappropriately concentrated urine, findings that overlap with syndrome of inappropriate antidiuretic hormone secretion (SIADH). However, suppressed adrenocorticotropic hormone and markedly reduced cortisol levels were consistent with secondary adrenal insufficiency.

Adrenocorticotropic hormone (ACTH) was below the detection limit, and serum cortisol was markedly reduced (0.96 μg/dL), consistent with secondary adrenal insufficiency in this clinical context [9]. Hypertonic saline was initiated.

Brain magnetic resonance imaging showed no hypothalamic or pituitary abnormalities (Figure 4). Pyuria worsened, and intravenous ceftriaxone was administered for 7 days, resulting in improvement.

On hospital day 14, intravenous hydrocortisone (350 mg/day) was initiated as stress‐dose therapy. The dose was gradually tapered according to clinical response and transitioned to oral hydrocortisone on hospital day 21. Following glucocorticoid replacement, her consciousness and serum sodium levels improved promptly, with no recurrence during follow‐up.

**FIGURE 2:**
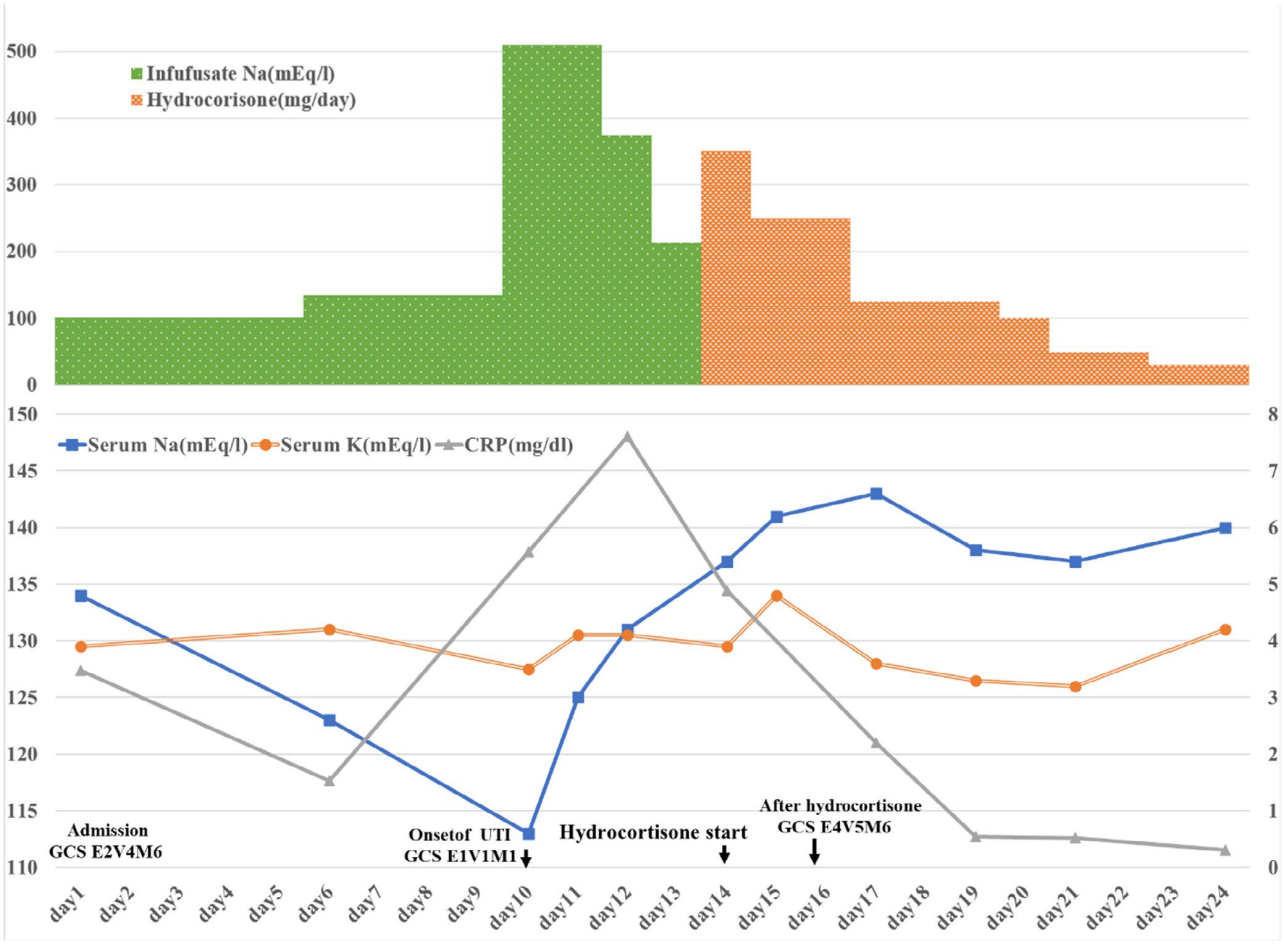
Clinical course during hospitalization. Changes in serum sodium concentration, C‐reactive protein, intravenous fluid sodium content, and hydrocortisone dose are shown. Glasgow Coma Scale (GCS) scores are indicated at major clinical events. Following initiation of hydrocortisone therapy, serum sodium levels and consciousness improved promptly. Primary axis: Serum sodium (mEq/L); secondary axis: Serum potassium (mEq/L) and CRP (mg/dL).

The clinical course is shown in Figure 2.

**FIGURE 3:**
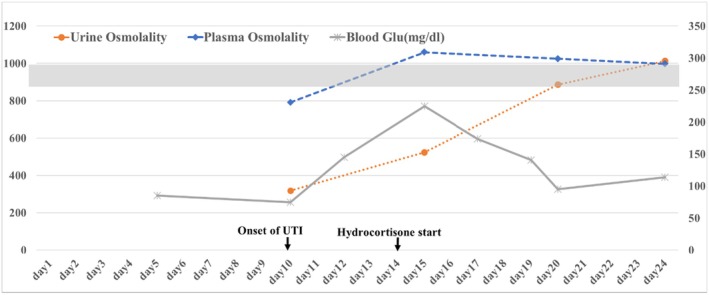
Changes in plasma osmolality, urine osmolality, and blood glucose during hospitalization. The shaded area indicates the normal range of plasma osmolality (275–295 mOsm/kg). Vertical dashed lines represent key clinical events. Primary axis: Urine osmolality. Secondary axis: Plasma osmolality and blood glucose.

Changes in plasma osmolality, urine osmolality, and blood glucose during hospitalization are shown in Figure 3. Despite hypotonic plasma osmolality, urine remained inappropriately concentrated, and hypoglycemia was also observed, suggesting adrenal insufficiency as a plausible contributing mechanism rather than primary SIADH.

The biochemical findings overlapped with those of syndrome of inappropriate antidiuretic hormone secretion (SIADH); however, the combination of suppressed ACTH, markedly low cortisol levels, and rapid response to hydrocortisone supported secondary adrenal insufficiency as a major contributing factor rather than primary SIADH alone.

## 4 Conclusion and Results (Outcome and Follow‐Up)

This case illustrates the possibility that severe hyponatremia in patients with long‐standing primary aldosteronism may be associated with suspected secondary adrenal insufficiency and possible cortisol autonomy.

Glucocorticoid replacement resulted in rapid resolution of severe hyponatremia and neurological impairment. The patient remained clinically stable without recurrence of hyponatremia under maintenance hydrocortisone therapy.

## 5 Discussion

This case highlights the potential clinical impact of possible unrecognized cortisol autonomy in patients with long‐standing primary aldosteronism (PA), particularly in the context of physiological stress.

Radiological stability of the adrenal tumor (Figure 1) did not reflect endocrine stability. Despite the absence of tumor progression over 13 years, the clinical course raised the possibility of chronic mild cortisol excess, which may have contributed to HPA axis suppression.

The possibility of secondary adrenal insufficiency was supported by undetectable ACTH, markedly reduced cortisol levels, hypotonic hyponatremia, and rapid clinical improvement following hydrocortisone administration. The clinical course (Figure 2) showed prompt recovery of both serum sodium levels and consciousness after glucocorticoid replacement, suggesting adrenal insufficiency as an important contributor to the clinical deterioration.

The pathophysiological features were further illustrated by changes in plasma and urine osmolality (Figure 3). Despite hypotonic plasma osmolality, urine remained inappropriately concentrated, mimicking syndrome of inappropriate antidiuretic hormone secretion (SIADH). However, the presence of hypoglycemia and the endocrine findings were not consistent with primary SIADH, suggesting adrenal insufficiency as the underlying mechanism.

Hypoglycemia may also have been influenced by reduced oral intake and antidiabetic treatment and should therefore be interpreted cautiously.

**FIGURE 4:**
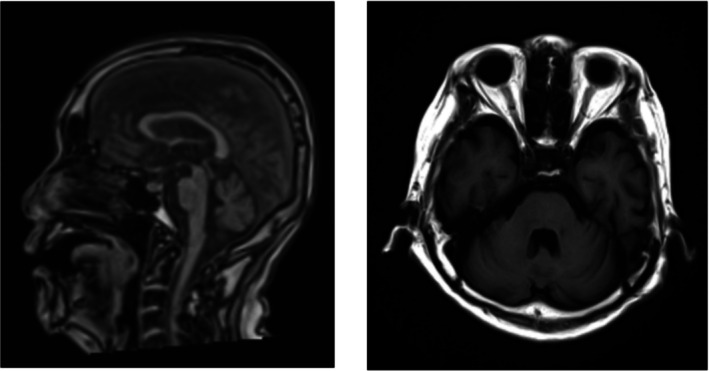
Brain magnetic resonance imaging showing no structural abnormalities of the hypothalamic–pituitary region. No evidence of pituitary or hypothalamic lesions was identified, suggesting the absence of a structural hypothalamic–pituitary cause.

In addition, brain magnetic resonance imaging (Figure 4) showed no structural abnormalities of the hypothalamic–pituitary region, suggesting the absence of a structural hypothalamic–pituitary cause.

Approximately 5%–10% of patients with PA reportedly exhibit concomitant ACS, including aldosterone‐ and cortisol‐producing adenomas (CAPA) [7, 8, 9, 10, 11, 12]. However, most evidence is derived from surgically treated cases. The natural history of conservatively managed PA with potential cortisol autonomy remains insufficiently characterized. This case underscores that radiological stability does not necessarily reflect endocrine stability.

A key clinical implication is the concept of a “subclinical but functionally fragile” endocrine state. Chronic mild hypercortisolism may maintain basal homeostasis while progressively suppressing ACTH secretion, resulting in impaired stress responsiveness. Under conditions such as infection or hospitalization, this latent vulnerability may manifest as overt adrenal insufficiency.

From a practical standpoint, clinicians should maintain a high index of suspicion for adrenal insufficiency in patients with PA who present with unexplained hyponatremia or clinical deterioration, even in the absence of overt Cushingoid features or tumor progression.

Limitations of this case include the absence of dynamic endocrine testing due to clinical instability. The diagnosis of cortisol autonomy could not be confirmed because dexamethasone suppression testing, urinary free cortisol measurement, late‐night cortisol assessment, and longitudinal endocrine evaluation were not performed. Likewise, adrenal reserve could not be formally assessed because ACTH stimulation testing was not feasible during the acute phase. In addition, surgical confirmation and adrenal venous sampling were not performed, and several potential contributors to hyponatremia, including infection, dehydration, and concomitant medications, could not be completely excluded. Therefore, the proposed pathophysiological association should be interpreted cautiously and regarded as hypothesis‐generating.

Taken together, the integration of clinical course, biochemical findings, and imaging suggests a possible association between secondary adrenal insufficiency and unrecognized cortisol autonomy in this patient with PA.

This case highlights a clinically important but potentially overlooked mechanism of hyponatremia in patients with primary aldosteronism.

## Author Contributions

**Hitomi Saito:** supervision. **Akito Maeshima:** supervision, writing – review and editing. **Honami Mori:** supervision. **Minami Toda:** data curation, writing – original draft, conceptualization, visualization, investigation, methodology. **Yutaka Kuroda:** supervision. **Shunsuke Takahashi:** supervision, writing – review and editing.

## Funding

The authors have nothing to report.

## Consent

Written informed consent was obtained from the patient or next‐of‐kin for publication of this case report and accompanying images.

## Conflicts of Interest

The authors declare no conflicts of interest.

## Data Availability

The data that support the findings of this study are available on request from the corresponding author. The data are not publicly available due to privacy or ethical restrictions.
